# Cytokine and satellite cell responses to muscle damage: interpretation and possible confounding factors in human studies

**DOI:** 10.1007/s10974-012-9303-z

**Published:** 2012-06-07

**Authors:** M. van de Vyver, K. H. Myburgh

**Affiliations:** Department of Physiological Sciences, Stellenbosch University, Private Bag X1, Matieland, Stellenbosch, 7602 South Africa

**Keywords:** Creatine kinase, Myoglobin, Pax7, Eccentric exercise

## Abstract

It is plausible that multiple muscle biopsies following a muscle damaging intervention can exacerbate the inflammatory and subsequent satellite cell responses. To elucidate confounding effects of muscle biopsy procedure on satellite cell number, indirect markers of damage and the inflammatory response following acute downhill running (DHR) were investigated. 10 healthy male participant were divided into a non-exercising control (*n* = 4) and DHR (12 × 5min bouts, 10 % decline at 85 % VO_2_max) (*n* = 6) group. Blood samples were taken pre, post and every 24 h for 9 days. Serum was analysed for creatine kinase (CK), myoglobin (Mb), lactate dehydrogenase (LDH), TNF-α, IL-6 and IL-10. Muscle biopsies taken on days 1 and 2 post intervention from opposing legs were analysed for Pax7^+^ satellite cells. In the DHR group, Mb (536 ± 277 ng mL^−1^), IL-6 (12.6 ± 4.7 pg mL^−1^) and IL-10 (27.3 ± 11.5 pg mL^−1^) peaked immediately post DHR, while CK (2651 ± 1911 U L^−1^), LDH (202 ± 47 U L^−1^) and TNF-α (25.1 ± 8.7 pg mL^−1^) peaked on day 1. A 30 % increase in Pax7^+^ satellite cells on day 1 in the DHR group was no longer apparent on day 2. H&E staining show evidence of phagocytosis in the DHR group. No significant changes over time were observed in the control group for any of the variables measured. Events observed in the DHR group were as a result of the intervention protocol and subsequent muscle damage. The relationship between SC proliferation and pro-inflammatory cytokine release appears to be complex since the IL-6/IL-10 response time differs significantly from the TNF-α response.

## Introduction

Exercise related studies still frequently use non-quantitative markers as indication that muscle damage occurred during exercise (Friden et al. 1989; Noakes 1987; Sorichter et al. 1999). The metabolic demands as well as the mechanical strain placed on muscle fibers during such unaccustomed exercise result in increased permeability of the sarcolemma, leading to the leakage of cytosolic muscle proteins (myoglobin (Mb), creatine kinase (CK), lactate dehydrogenase (LDH), skeletal troponin I and myosin heavy chain) into the circulation (Friden et al. 1989; Jamurtas et al. 2005; Melin et al. 1997; Newham et al. 1983a; Nosaka and Clarkson 1996; Sayers and Clarkson 2003; Sorichter et al. 1997, 1999). In addition to sarcolemmal damage, the mechanical strain during muscle damaging exercise can cause abnormal movement of the basement membrane (Kyrolainen et al. 1998; Lauritzen et al. 2009). This is sufficient to trigger the activation of satellite cells (Moss and LeBlond 1970) that in turn play an important role in the repair of the damaged muscle (Hawke and Garry 2001).

The number of activated satellite cells per muscle fiber may be used as an indication of proliferation in response to the muscle-damaging exercise (Moss and LeBlond 1970; Muir et al. 1965). Early studies in animal models have more recently been followed up by studies in human subjects subjected to resistance exercise interventions (Dreyer et al. 2006; Lauritzen et al. 2009; Malm et al. 2000). Acute responses in Pax7^+^ satellite cell (Pax7^+^ SC) number have been assessed using different methods (McKay et al. 2010), yielding consistent results. However, if multiple muscle biopsies are taken after the damaging exercise, the possibility exists that a response to the first biopsy may exacerbate the number of satellite cells activated in subsequent biopsies. This would render the conclusions on the satellite cell responses to damaging exercise itself invalid. Even if the second biopsy is taken from the opposite leg, it is conceivable that a circulating inflammatory effect may influence satellite cell number (Li Yi Ping 2003; Malm et al. 2000).

On the other hand, similar to possible effects on circulating markers of muscle damage, the needle biopsy procedure may cause confounding effects when investigating the inflammatory response to damaging exercise. Not only could the inflammatory response be exacerbated, but the resultant peak concentrations for the circulating indicators could change and their resolution could also be delayed. Typically TNF-α and IL-6 are used as cytokines indicating the extent of the inflammatory response (pro-inflammatory), whilst IL-10 counteracts IL-6. However, indirect serum markers do not indicate the extent of muscle structural damage and it needs to be questioned whether muscle biopsy sampling super-imposed on the time frame following the damaging exercise influences the extent or time course of appearance of these markers. There is thus a necessity for human exercise models to explain skeletal muscle plasticity in health and injury.

Nonetheless, a biopsy is a relatively small and localised area, whereas the exercise-induced damage is diffuse. Therefore, we hypothesise that circulating markers of muscle damage will not be affected by two needle biopsy procedures separated by 1 day. Satellite cells are responsive to basement membrane damage as well as the molecular influences, therefore we hypothesise that the number of satellite cells will increase in biopsies taken on consecutive days, despite significant difference in location of the second biopsy.

The specific aims of this study included the assessment of satellite cell number, the measurement of commonly used indirect markers of damage as well as the inflammatory response, both assessed on a daily basis over a period of 9 days. A major aim was to elucidate any possible confounding effect of the biopsy procedure by including both a downhill running (DHR) group and non-exercising control group.

## Materials and methods

### Subjects

Ten healthy young men volunteered to participate in this study and were randomly subdivided into a control (*n* = 4) and DHR group (*n* = 6). All of the participants were unaccustomed to any form of downhill activity and reported no exercise related injury within the previous 3 months. Participants were informed about the purpose and risks of the study before signing an informed consent document. The experimental protocol was approved by the Committee for Human Research at Stellenbosch University and the study was conducted according to the ethical guidelines and principles of the International Declaration of Helsinki.

### Exercise testing

#### Incremental VO_2_max test

Following a 5 min warm-up on the treadmill (Runrace, Technogym, Italy), all participants were asked to wear an oxygen mask linked to a metabolic analysis system (Oxycon Pro, Jaeger, Germany) for breath-by-breath gas analysis. The test started at 8 km h^−1^ for 30 s after which the speed increased 0.5 km h^−1^ every 30 s until exhaustion. Participants were verbally encouraged to continue as long as possible.

#### Downhill run

The participants within the DHR group performed a 60-minute intermittent DHR (12 × 5 min bouts at 85 % VO_2_max, 10 % decline) on the same motorized treadmill. They were allowed a 2 min standing rest between bouts and all the participants were able to complete all twelve bouts.

### Muscle soreness

Participants were asked to self-assess the muscle soreness they experienced using a scale from 1 to 5, with 1 associated with no pain and 5 associated with very severe pain. Muscle soreness was assessed immediately before and on days 1, 2 and 9 following the DHR.

### Blood sampling

Blood was drawn from the antecubital vein in the supine position immediately before (pre) and post DHR and every morning for 9 days (day 1–9). Blood samples were left to coagulate in the tubes and then centrifuged at 3,000 rpm for 10 min at 4 °C, serum was frozen at −80 °C for later analysis. All serum samples were analyzed using one-step sandwich assays for creatine kinase activity (CARDIAC Calibrator # 386371), myoglobin concentration (CARDIAC Calibrator # 973243), and lactate dehydrogenase activity (SYNCHRON CX SYSTEM, kit # 443793). The pre, post, days 1, 2, 3, 5, 6 and 9 serum samples of the DHR group were also analyzed for inflammatory cytokines (TNF-α, IL-6, IL-10) using a plexed suspension array system (Bio-Plex, BioRad Laboratories). The lowest level for detection for the various cytokines were as follows: TNF-α limit 0.24 pg mL^−1^, IL-6 limit 0.3 pg mL^−1^, IL-10 limit 0.38 pg mL^−1^.

### Muscle biopsy

Muscle biopsies were obtained from the *vastus lateralis* muscle using a 5 mm trephine biopsy needle with assisted suction. Four biopsies were obtained from each subject; 6 weeks before (right leg) the DHR, day 1 (left leg), day 2 (right leg) and day 9 (left leg) post exercise with a distance of 3 cm between biopsy sites on the left leg. The biopsies were placed in a 4 % formalin saline solution and embedded in paraffin wax for immunofluorescence microscopy.

### Laboratory analysis

5 μm microtome (CM1100, Leica Microsystem, Germany) sections were made from the paraffin wax embedded biopsy samples.

#### Haematoxylin and eosin staining

Sections were incubated in xylene (2 × 10 min) followed by dehydration in different concentrations of alcohol (100, 95, 80, 50 % with10 dips in each). Following the dehydration sequence sections were rinsed in distilled H_2_O before being placed in Harris hematoxylin solution for 3 min. Sections were once again rinsed in distilled H_2_O and placed briefly in 1 % acid alcohol solution. This was followed by an incubation step using Scott’s tap water and then sections were placed in Eosin working solution (2 min). After a final rinsing step, sections were once again placed in different concentrations of alcohol (70, 95,100 % with 10 dips in each) and finally dipped in xylene. Sections were immediately mounted using synthetic mounting medium (Shandon # 67690007).

#### Immunohistochemistry

Sections from the paraffin embedded muscle biopsy samples were incubated in xylene (2 × 5 min) followed by dehydration in different concentrations of alcohol (100 %, 2 × 3 min; 95 %, 2 × 3 min; 80 %, 3 min; 50 %, 3 min). Sections were washed in PBS and incubated with 20 % goat blocking serum for 30 min at room temperature. This was followed by an overnight incubation at 4 °C with anti-Pax7 antibodies [1:50] (mouse monoclonal, Hybridoma bank, Iowa). Sections were then washed with PBS and the secondary antibody [1:200] (Invitrogen, Alexa Fluor 488 goat anti-mouse IgG) administered in the dark for 60 min. This was followed by another washing step and sections were then incubated with anti-Laminin antibodies [1:250] (DAKO, polyclonal rabbit anti-laminin #00037479) in the dark for 4 h. Sections were once again washed and incubated with the next secondary antibody [1:200] (Invitrogen, Alexa Fluor 594 goat anti rabbit) for 60 min in the dark followed by another washing step with PBS. Sections were then incubated with [1:200] Hoescht/4′6-diamidino-2phenylindole for 15 min and mounted using fluorescent mounting medium (DAKO, USA). Pictures were taken under a fluorescent microscope (Eclipse E400, Nikon) and analysed using Image J software.

### Statistical analysis

All data are presented as mean ± SEM. StatSoft (STATISTICA 9 version 2 and InStat) software was used for all statistical analysis. two-way Factorial ANOVA with Fisher’s post hoc test was used to determine significant Group × Time effects. Repeated measures ANOVA with Newman Keuls post hoc test was used to determine significant changes over time within each group separately. Because of the small sample size multiple regression analysis could not be performed, instead Pearson’s linear correlation analysis was used to determine whether significant relationships existed between variables. Because of the number of parameters in this analysis, a Bonferroni correction was used and adjusted *r* ^2^ values reported.

## Results

### Subject characteristics

Mean ± SE: age 22 ± 0.2 year, BMI 23.7 ± 0.9 kg m^−2^, VO_2_max 52.4 ± 1.9 mL kg min^−1^.

### Delayed onset muscle soreness

The control group experienced no pain (rating of 1 ± 0). All individuals in the DHR group experienced severe soreness (rating of 4 ± 0) on day 1 with continued soreness on day 2. Therefore, there was a significant group effect (two-way factorial ANOVA, *p* < 0.001) for both days. In the DHR group, individual variation was evident on day 2 ranging from moderate (rating of 3) to very severe (rating of 5) pain.

### Hematoxylin & eosin staining

**Fig. 1:**
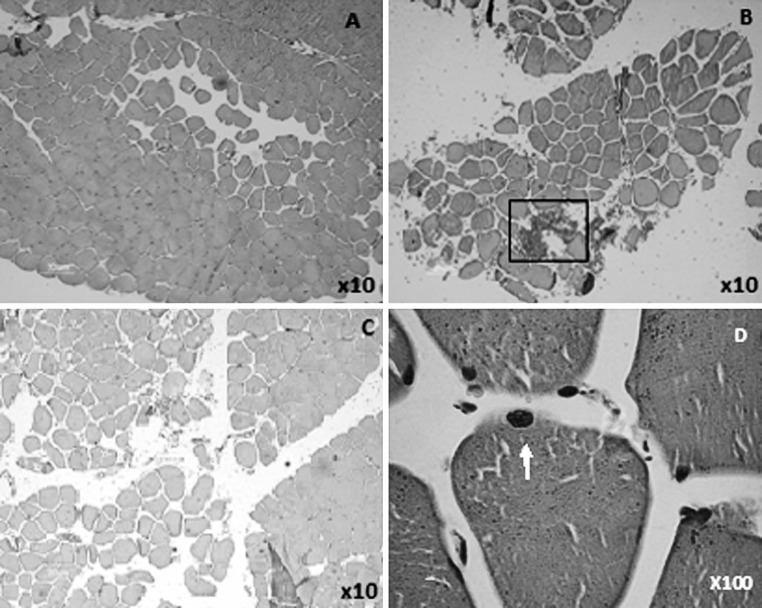
Hematoxylin and eosin stain. **a** Baseline. **b** Day 1. **c**, **d** Day 2. Pictures are from one subject representative of the overall effect observed within the DHR group. The *block* indicates an area of *red* cell invasion. *Arrow* indicates area of damage to the basal lamina in a single fibre (**d**). *DHR* downhill running. (Color figure online)

A typical example of H&E staining from a baseline biopsy is presented in Fig. 1a and indicates expected normal muscle fiber arrangement. On day 1 and day 2 red blood cells can be observed between fibers and muscle fibers seemed to be further apart suggesting that some swelling had taken place (Fig. 1b, c). At higher magnification (×40) evidence of basal lamina damage can be observed on day 2 (Fig. 1d).

### Indirect indicators of muscle damage

**Fig. 2:**
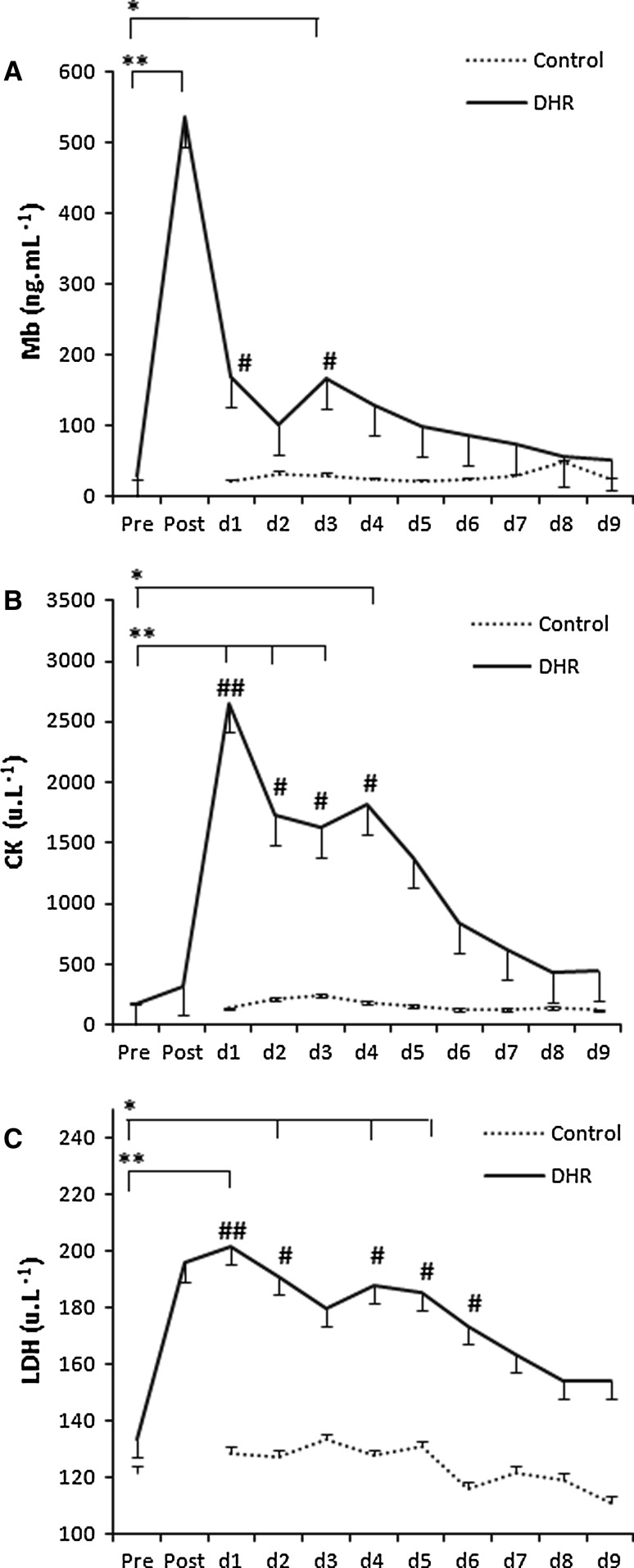
Indirect markers of muscle damage: DHR vs. control group. **a** Myoglobin. **b** Creatine kinase. **c** Lactate dehydrogenase. (mean ± SE). **p < 0.001, *p < 0.01 indicate time points significantly different from the pre-exercise value in the DHR group (*n* = 6). ^##^ *p* < 0.001, ^#^ *p* < 0.01 indicate significant difference between groups evident at the same time point after DHR. Statistical analysis: two-way factorial ANOVA with Fisher’s post hoc test (Group effect and Time effect). Footnote: biopsies were taken on day 1, 2 and 9 for both groups

**Fig. 3:**
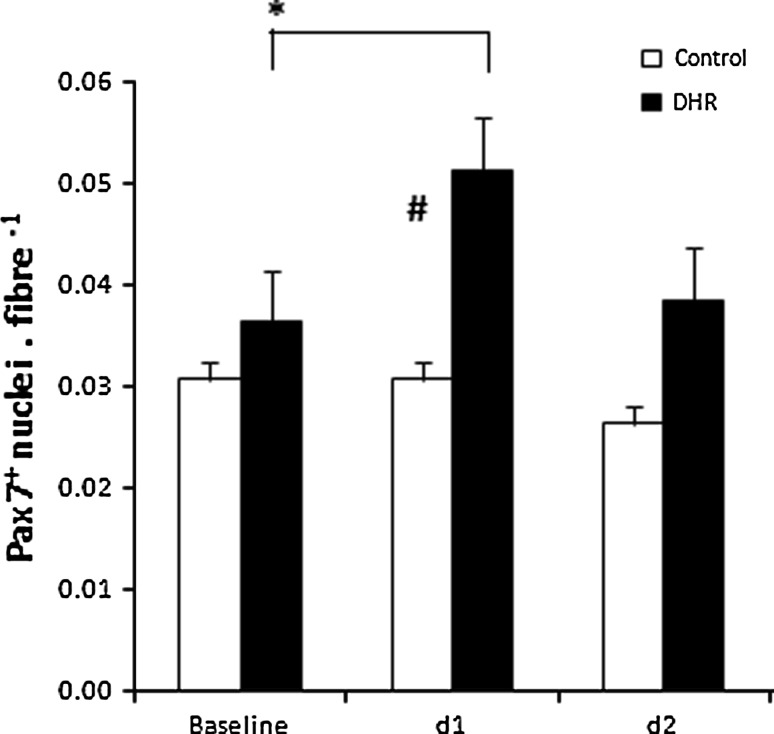
Pax7^+^ satellite cell nuclei per fibre (mean ± SE). *p < 0.02 time points significantly different from the pre value in the DHR group (*n* = 6). For the control group (*n* = 4) statistical analysis indicated no effect over time. ^#^ *p* < 0.01 significant difference between groups at the same time point. Statistical analysis: two-way factorial ANOVA with Fisher’s post hoc test (group by time effect)

In the DHR group, Mb concentration peaked immediately post exercise (536 ± 113 ng mL^−1^), whereas the CK (2651 ± 780 U L^−1^) and LDH activities (202 ± 19 U L^−1^) peaked on day 1 and remained elevated for 4–6 days (Fig. 2). In the control group, at the same time points as above, the myoglobin concentration (Mb) (33 ± 3 ng mL^−1^), creatine kinase activity (CK) (247 ± 38 U L^−1^) and lactate dehydrogenase activity (LDH) (128 ± 9 U L^−1^) (mean ± SE) remained within the normal reference range. A group effect as well as a time effect was observed although post hoc differences were not the same for each indirect marker of damage (Fig. 3).

### Pro- and anti-inflammatory cytokines

Immediately post exercise the serum concentrations of the pro-inflammatory cytokine IL-6 and the anti-inflammatory cytokine IL-10 were significantly higher than the pre values (*p* < 0.05) in the DHR group (Table 1). TNF-α concentrations presented with a delayed response and were significantly elevated on day 1 (*p* < 0.01) following exercise (Table 2).

**Table 1:** Individual data (DHR group): peak circulating markers of muscle damage

Subject (#)	Mb (post)	CK (day 1)	LDH (day l)
001	344	1198	166
002		866	148
003	631	2401	223
004	391	3115	197
005	332	2147	194
006	982	6177	282

Values are presented as Mb (ng mL^−1^), CK (U L^−1^), LDH (U L^−1^)

**Table 2:** Serum cytokine concentrations (mean ± SE) observed in the DHR group over time

	Pre	Post	Day 1	Day 2	Day 3	Day 5	Day 6	Day 9
IL-6	3.4 ± 1.5	12.6 ± 4.7^#^	9.6 ± 2.0	4.5 ± 1.3	2.8 ± 0.9	2.1 ± 0.9	1.6 ± 0.9	3.7 ± 1.1
IL-10	7.8 ± 2.3	27.3 ± 11.5^#^	18.4 ± 3.7	12.7 ± 3.6	5.6 ± 1.4	5.6 ± 1.4	3.8 ± 1.4	9.9 ± 3.1
TNF-α	9.2 ± 3.6	8.8 ± 3.6	25.1 ± 8.7*	5.6 ± 2.7	4.4 ± 1.9	5.3 ± 2.2	2.1 ± 0.8	9.0 ± 3.9

Values (mean ± SE) are given in ng mL^−1^. Statistical analysis: repeated measures ANOVA, Newman Keuls post hoc test

* *p* < 0.01, ^*#*^ *p* < 0.05 indicate time points significantly different from the pre- value in the DHR group

### Satellite cell count

**Fig. 4:**
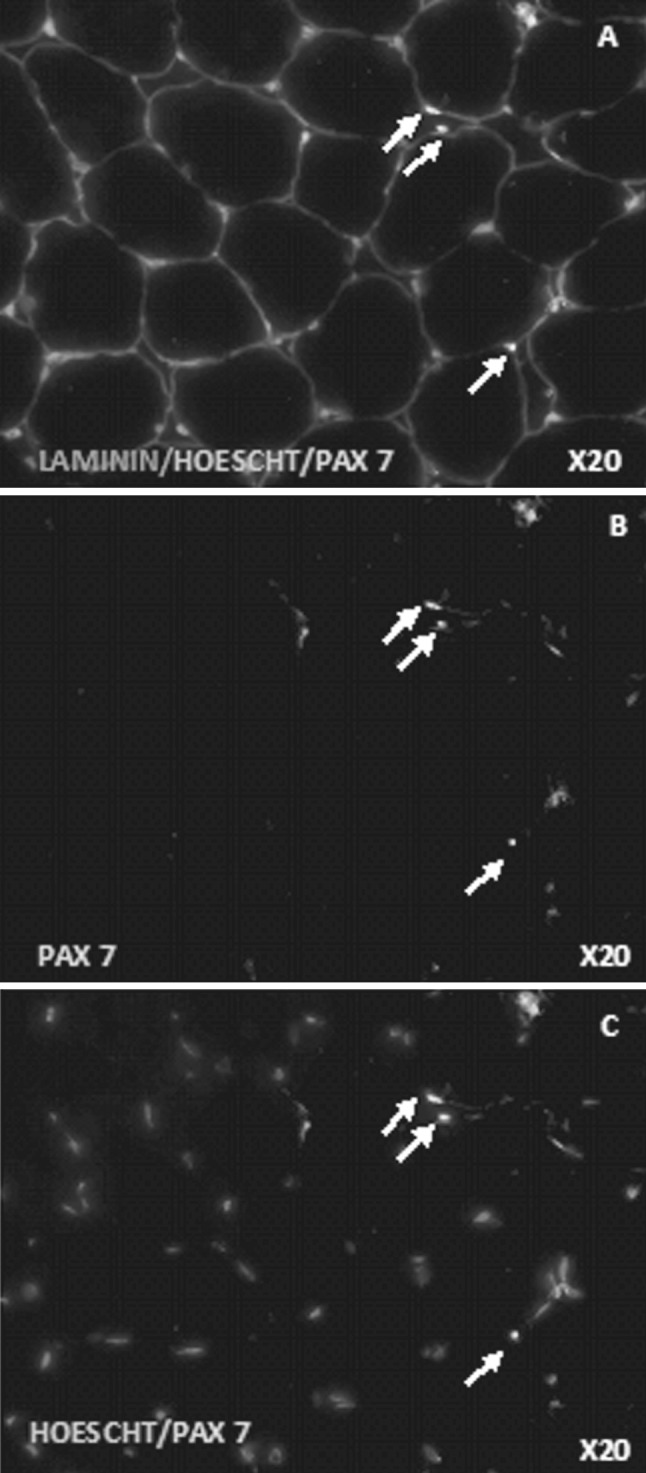
Immunohistochemistry: Pax7/Laminin double stain. Representative fluorescent microscopy images. **a** Co-stain anti-laminin (*red*), anti-Pax7 (*green*) and Hoescht (*blue*) **b** anti-Pax7 (*green*), **c** co-stain Hoescht (*blue*) and anti-Pax7 (*green*). All the nuclei are stained by Hoechst (*blue*), the quiescent and proliferating satellite cell nuclei are stained by both Hoescht (*blue*) and anti-Pax7 (*green*), the basal lamina is stained by anti-laminin (*red*). *Arrows* indicate Pax7^+^ satellite cell nuclei. (Color figure online)

Compared to baseline there was a significant (*p* < 0.02) increase in the number of Pax7^+^ cells per fiber (Fig. 4) only on day 1 post exercise in the DHR group whereas no change over time was observed for the control group (Fig. 3).

## Discussion

This study shows that an acute DHR protocol is as effective as acute resistance exercise in inducing satellite cell proliferation within 1 day. We also provide evidence that this proliferation was not sustained, since SC number returned to baseline levels after one more day. Plausible explanations for this finding are that the pro-inflammatory cytokines which have proliferative effects, both IL-6 and TNF-α, were elevated only in the early phases post-DHR. A second important finding was that the IL-6 response to DHR resolved very quickly whilst the TNF-α elevation occurred a day later, suggesting alternative mechanisms. Finally, the two muscle biopsies taken on consecutive days after DHR (day 1 and day 2) did not influence the validity of elevations in circulating markers of muscle damage or the significant elevation in satellite cell numbers. The use of the control group who underwent only the muscle biopsies allowed for these definitive conclusions.

Although H&E staining is not as accurate as electron microscopy to indicate signs of sarcomere and mitochondrial damage, open spaces between muscle fibers could be clearly observed on day 1 and day 2 suggesting that edema was present between individual fibers of the *vastus lateralis* muscle of the DHR group for at least the first 48 h following exercise. There was some evidence of damage to the basal lamina, which may have been induced by phagocytosis (Armstrong et al. 1991). Roth et al. (2000) suggested that muscle biopsy samples should be viewed with caution when quantifying muscle damage, since the needle biopsy procedure and processing itself can result in muscle damage, specifically hypercontraction of fibers. After reviewing the literature Roth et al. (2000) concluded that hypercontracted fibers in vivo are usually accompanied by signs of necrosis whereas hypercontracted fibers without necrosis are the result of the biopsy procedure. Signs of necrosis are however not frequently seen in muscle damaged by eccentric exercise without electrical stimulation or forced lengthening. Normal skeletal muscle structure was evident in the baseline biopsies and in the control subjects’ biopsies at all the time points. Therefore, it is likely that the abnormalities observed in the biopsies of the DHR subjects on day 1 and day 2 are the result of the DHR and not the biopsy procedure. Other convincing evidence that damage can be observed without electron microscopy is that significant red cell invasion was seen in several areas of edema (Marqueste et al. 2008) rather than hypercontraction or biopsy artefact. The descriptive nature of these observations could, in future be quantified. We hypothesise that red cell invasion and fiber separation may vary between subjects and possibly explain differences in the peak DOMS response or the variability in DOMS that was seen after 48 h.

The presence of severe muscle damage is associated with satellite cell proliferation (Buford et al. 2009; Jamurtas et al. 2005) and an increased number of satellite cells has been reported within the first 24 h following an acute bout of eccentric resistance exercise (Dreyer et al. 2006). However, Malm et al. (2000) indicated that 30 min of eccentric cycling combined with multiple biopsies or the multiple biopsies without exercise (7 biopsies over a period of 7 days) resulted in a similar satellite cell (CD56^+^cell count) response. Despite the immune changes that the authors observed in both groups, DOMS and CK levels remained unchanged in the group subjected to multiple biopsies alone. In the present study the satellite cell numbers remained unchanged in the control group on day 1 and day 2, suggesting that only two muscle biopsies are insufficient to contribute to any change in Pax7^+^ cells. Other studies have shown that there are small differences in satellite cell count when identified using Pax7 vs. CD56 (Mackey et al. 2009), but the discrepancy between these markers is very small. In the present study the 2 needle biopsies were taken from opposing legs, whereas Malm et al. (2000) had taken at least 3 muscle biopsies from each leg over a relatively short period of time. Therefore, any change in Pax7^+^ cells observed in the DHR group could be ascribed to DHR itself. Indeed, the number of Pax7^+^ cells observed in the DHR group was substantially increased (+30 %). It can be concluded that two muscle biopsies had no confounding effect, although the possibility still exists that two or more biopsies taken from the same leg might influence the magnitude of satellite cell activation and proliferation.

Most investigations into the mechanism of eccentric exercise-induced muscle injury support a mechanical origin of damage (Armstrong et al. 1991; Friden et al. 1989; Lauritzen et al. 2009; Lieber and Friden 1999; Morgan 1990; Morgan and Allen 1999; Newham et al. 1983a, b; Raadstad et al. 2010). The mechanical strain results from the lengthening of sarcomeres while they are contracting (Morgan 1990; Morgan and Allen 1999; Raven 1991), which is proposed to result in *Z*-line streaming and sarcomeric protein disruption (Armstrong 1984; Armstrong et al. 1991; Friden et al. 1989; Lieber and Friden 1999; Morgan 1990). The exercise protocol in this study was designed based on observations of Eston et al. (1995). DHR activates a combination of eccentric and concentric muscle contractions and is a less localised eccentric stimulus than that applied in models using biceps extension or knee extensions or other forms of resistance exercise. Nonetheless, severe muscle soreness hindered the normal daily activities of subjects in the DHR group and coincided with significant increases in serum markers of damage, with peak serum CK activity within the first 24 h post exercise. In severe cases of lengthening contractions, damage to the t-tubules and sarcolemma occur (Lauritzen et al. 2009; Morgan and Allen 1999; Newham et al. 1983a; Takekura et al. 2001). The membrane damage has been hypothesised to exacerbate the leakage of muscle proteins into the circulation. The current study presents qualitative evidence of immune cell attraction and invasion, as well as phagocytosis including membrane disruption (see insert in Fig. 1b). It remains to be definitively proven whether or not rupturing of the sarcolemma is part of the primary damage, or whether it is evidence of secondary damage induced by phagocytic immune cells.

The inflammatory response to damaging exercise is in addition to the inflammatory response to exercise without damage. A significant IL-6 response was evident immediately after DHR, thus IL-6 production occurred during exercise. The extent of increase was approximately threefold that seen from a concentric cycling protocol lasting 60 min (means of 12.6 vs. 4.5 pg mL^−1^) (MacDonald et al. 2003). This would suggest that DHR results in a greater release of IL-6 into the circulation. However, the IL-6 release was equivalent to that seen after a 3 h cycling protocol (Penkova et al. 2003), indicating that the IL-6 response was unlikely due to the eccentric nature of the exercise alone. Of greater importance in the context of the current study is that the pro-inflammatory IL-6 response was tightly linked to an anti-inflammatory process. Specifically, the DHR protocol also resulted in an IL-10 response that was double that of IL-6. Previously, an infusion of rhIL-6 at rest has resulted in a robust IL-10 response (Steensberg et al. 2003). This raises the question of whether or not IL-6 is involved in satellite cell proliferation in the in vivo context of exercise. The IL-6 superfamily includes LIF (leukemia inhibiting factor) which is unequivocally known to affect satellite cell proliferation (Hawke and Garry 2001). The significant increase in Pax7^+^ cells occurred 24 h after the peak IL-6, and rather coincided with peak TNF-α, also a cytokine known to influence satellite cell proliferation (Li Yi Ping 2003). There was individual variability in both IL-6 and TNF-α, but the Pax7^+^ cell count response was considerable in all subjects.

Individual variability also existed in the markers of muscle damage, ranging from 866 to 6,177 U L^−1^ (peak CK), 148–282 U L^−1^ (peak LDH) and 332–982 ng mL^−1^ (peak Mb). This range indicates that at least one subject experienced exertional rhabdomyolysis. Yamin et al. (2008) recently reported that subjects with an IL-6 gene polymorphism (with SNP specifically at 174CC) had a more than threefold statistical probability of experiencing a very high CK release after eccentric exercise. The TNF-α genotype of the same subjects showed only a trend toward affecting CK response. This is interesting, because the IL-6 response is so rapidly countered by the anti-inflammatory cytokine IL-10 and the TNF-α peak is more closely associated with the time point of CK peak.

The time courses of resolution differed considerably for the different indirect markers of muscle damage measured, a finding that is not a new observation. The fact that CK and LDH activities remain elevated for several days, implies either a longer duration of release than myoglobin for example, or slower clearance from the circulation, or a combination. Based on a review of the literature, Brancaccio et al. (2007) suggested that the time course of CK release into and clearance from the circulation might be dependent on the level of training. In agreement with this suggestion, VO_2_max correlated strongly with peak values of all three markers, CK (*r* ^2^ = 0.706), Mb (*r* ^2^ = 0.8458) and LDH (*r* ^2^ = 0.7869). Another suggestion that has been made is that the time course of appearance is dependent on the size of the molecule, since small proteins such as myoglobin (17 kDa) are released directly into the circulation whereas larger proteins such as CK (80 kDa) are taken up by the lymphatic system before entering the blood stream (Lindena and Kupper 1979). This has been supported by Sayers and Clarkson (2003) who indicated a blunted CK response following eccentric exercise of the elbow flexors when the exercising arm was immobilized compared to a control non-immobilized group. Muscle movement has an effect on lymph flow and therefore may affect CK release into the blood (Sayers and Clarkson 2003).

## Conclusion

Since no significant changes over time were observed in the control group for any of the variables measured, it can be concluded that the events observed in the DHR group were as a consequence of the intervention protocol and subsequent muscle damage. This finding is important because it allows for future experimentation on the integration between the nature of muscle damage, the inflammatory response and the subsequent satellite cell proliferation to be investigated with doubt that the findings could be assigned to a biopsy response. This is also the first study to show that a relatively simple whole body exercise such as DHR is sufficient to induce a similar extent of SC proliferation compared with more extreme eccentric exercise of isolated muscle groups. The relationship between SC proliferation and pro-inflammatory cytokine release appears to be complex since the IL-6/IL-10 response time differs significantly from the TNF-α response.
